# A subset of sweet-sensing neurons identified by IR56d are necessary and sufficient for fatty acid taste

**DOI:** 10.1371/journal.pgen.1007059

**Published:** 2017-11-09

**Authors:** John M. Tauber, Elizabeth B. Brown, Yuanyuan Li, Maria E. Yurgel, Pavel Masek, Alex C. Keene

**Affiliations:** 1 Department of Biological Sciences, Florida Atlantic University, Jupiter, FL, United States of America; 2 Department of Biological Sciences, Binghamton University, Binghamton, NY, United States of America; Katholieke Universiteit Leuven, BELGIUM

## Abstract

Fat represents a calorically potent food source that yields approximately twice the amount of energy as carbohydrates or proteins per unit of mass. The highly palatable taste of free fatty acids (FAs), one of the building blocks of fat, promotes food consumption, activates reward circuitry, and is thought to contribute to hedonic feeding underlying many metabolism-related disorders. Despite a role in the etiology of metabolic diseases, little is known about how dietary fats are detected by the gustatory system to promote feeding. Previously, we showed that a broad population of sugar-sensing taste neurons expressing Gustatory Receptor 64f (*Gr64f*) is required for reflexive feeding responses to both FAs and sugars. Here, we report a genetic silencing screen to identify specific populations of taste neurons that mediate fatty acid (FA) taste. We find neurons identified by expression of Ionotropic Receptor 56d (*IR56d*) are necessary and sufficient for reflexive feeding response to FAs. Functional imaging reveals that IR56d-expressing neurons are responsive to short- and medium-chain FAs. Silencing IR56d neurons selectively abolishes FA taste, and their activation is sufficient to drive feeding responses. Analysis of co-expression with Gr64f identifies two subpopulations of IR56d-expressing neurons. While physiological imaging reveals that both populations are responsive to FAs, IR56d/Gr64f neurons are activated by medium-chain FAs and are sufficient for reflexive feeding response to FAs. Moreover, flies can discriminate between sugar and FAs in an aversive taste memory assay, indicating that FA taste is a unique modality in *Drosophila*. Taken together, these findings localize FA taste within the *Drosophila* gustatory center and provide an opportunity to investigate discrimination between different categories of appetitive tastants.

## Author summary

Fat represents a calorically potent food source that yields approximately twice the amount of energy as carbohydrates or proteins per unit of mass. Dietary lipids are comprised of both triacylglycerides and FAs, and growing evidence suggests that it is the free FAs that are detected by the gustatory system. The highly palatable taste of FAs promotes food consumption, activates reward centers in mammals, and is thought to contribute to hedonic feeding that underlies many metabolism-related disorders. Despite a role in the etiology of metabolic diseases, little is known about how dietary fats are detected by the gustatory system to promote feeding. We have identified a subset of sugar-sensing neurons in the fly that also responds to medium-chain FAs and are necessary and sufficient for behavioral response to FAs. Further, we find that despite being sensed by shared neuronal populations, flies can differentiate between the taste of sugar and FAs, fortifying the notion that FAs and sugar represent distinct taste modalities in flies.

## Introduction

Fat represents a calorically potent food source that yields approximately twice the amount of energy as carbohydrates or proteins per unit of mass. In mammals, dietary lipids are detected by taste cells, mechanosensory and olfactory neurons, as well as by post-ingestive feedback [1–4]. Dietary lipids are comprised of triacylglycerides and FAs, and growing evidence suggests that it is the free FAs that are detected by the gustatory system [5–7]. Sensing of FAs promotes food consumption, activates reward circuitry, and is thought to contribute to hedonic feeding that underlies many metabolism-related disorders [8,9]. Despite a role in the etiology of metabolic diseases, little is known about how dietary fats are detected by the gustatory system to promote feeding.

In flies and mammals, tastants are sensed by dedicated gustatory receptors that localize to the taste cells or taste receptor neurons [10–12]. These cells are sensitive to different taste modalities such as sweet, bitter, salty, sour, or umami, and project to higher order brain structures for processing [10,13,14]. While these taste modalities have been extensively studied, much less is known about how FAs are detected and how this sensory stimulus is processed. Taste neurons in *Drosophila* are housed in gustatory sensilla located on the tarsi (feet), proboscis (mouth), and wings. Each sensillum contains dendrites of up to four gustatory receptor neurons (GRNs), which are activated by different categories of tastants [15]. Two main classes of neurons include one group that senses sweet tastants and promotes feeding, and a second, non-overlapping group that senses bitter tastants and promotes avoidance [16,17]. We previously showed that *Drosophila* is attracted to medium-chain FAs [18]. Consumption of FAs relies on taste, rather than smell, as it is not impaired by surgical ablation of olfactory organs [18]. Additionally, FA consumption is abolished in *pox-neuro* mutants in which all external taste hairs are converted to mechanosensory bristles, indicating that the chemical signature rather than oily texture of FAs is associated with perception [18]. Silencing of Gr64f-expressing neurons, which are required for sugar sensing [19,20], also abolishes behavioral responses to FAs, suggesting that shared populations of gustatory neurons detect FAs and sugars [18]. Unlike sugars, FA sensing is dependent on functional Phospholipase C (PLC), suggesting that independent intracellular molecular signaling regulates FA and sugar taste [18]. However, any further characteristics of the physiological response or the specific neuronal identity of the neurons mediating FA response are unknown.

Taste neurons from the legs and proboscis predominantly project to the subesophageal zone (SEZ), the primary taste center of the *Drosophila* central nervous system, but the downstream central brain circuitry contributing to taste processing is less well understood [21–24]. Determining how diverse tastants activate GRNs that convey information to the SEZ, and how this information is represented in higher order brain centers, is central to understanding the neural basis for taste processing and feeding behavior. Identifying the neural principles underlying FA taste processing requires localizing FA-responsive taste neurons and characterizing their innervation of the primary taste center. Recent studies in *Drosophila* have identified taste neurons that are responsive to diverse modalities including salt, sugar, amino acids, water, carbonation, bitter, polyamines, and electrophilic tastants [16,25–31], yet little is known about the populations underlying FA taste. Here, we show that GRNs identified by expression of the *IR56d*, which partially overlap with Gr64f-expressing neurons, are necessary and sufficient for the feeding response induced by medium-chain FAs. Our results reveal a defined population of neurons that sense FAs to promote food consumption, providing a mechanism for differentiation between attractive tastants of different modalities.

## Results

**Fig 1:**
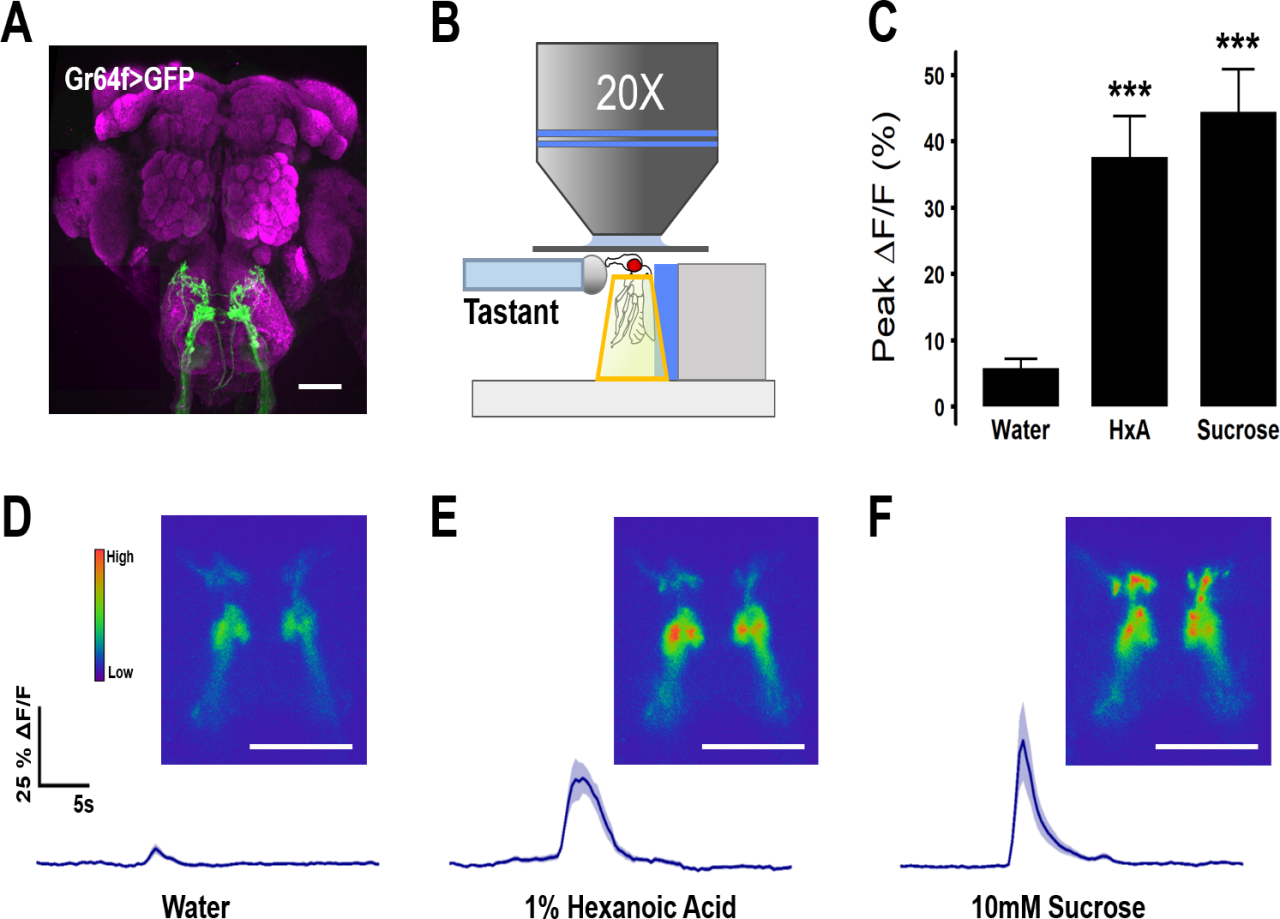
Gr64f gustatory receptor neurons respond to sucrose and HxA. (A) Expression of GFP in Gr64f-GAL4 neurons reveals axon terminals in the sub-esophagael zone (SEZ). Scale bar = 50 μm. (B) Diagram of live-imaging experimental protocol. Cuticle above the SEZ is removed and GCaMP5 fluorescence is recorded while tastant is applied to the proboscis. (C) Average peak %ΔF/F during response to water, 1% HxA, and 10mM sucrose (n = 11, 11, 10 respectively). Error bars indicate SEM. One-way ANOVA with Tukey’s HSD; ***p<0.001. (D) Average %ΔF/F traces and representative images of calcium activity in Gr64f neurons responding to water, (E) 1% HxA, and (F) 10mM sucrose. Scale bar = 50 μm. Shaded region of trace indicates +/- SEM.

We previously reported that silencing Gr64f-expressing taste neurons abolishes behavioral responses to both sugars and FAs [10]. To directly investigate the responsiveness of these neurons to FAs, we expressed the Ca^2+^ sensor GCaMP5 under control of Gr64f-GAL4 [32,33] (Fig 1A). The Ca^2+^ responses to proboscis application of water, sucrose, or the medium-chain FA, hexanoic acid (HxA), were monitored *in vivo* in the axonal projections of Gr64f neurons within the SEZ (Fig 1B). Flies were provided either with 10mM sucrose or 1% HxA because these concentrations induce comparable levels of Proboscis Extension Reflex (PER) behavior. Both 10mM sucrose and 1% HxA induced activity in the SEZ, while little response was observed to water alone (Fig 1C–1F). The temporal dynamics of Ca^2+^ activity differed between the two tastants, with HxA eliciting a longer lasting response (Fig 1E and 1F), yet both elicited comparable peak changes in fluorescence (Fig 1C). Therefore, both sugars and FAs activate Gr64f-expressing, sweet-sensing GRNs, fortifying the notion that this neuronal class is generally responsive to attractive tastants.

To localize FA-sensitive neurons within the broad Gr64f population, we selectively silenced neuronal populations predicted to overlap with Gr64f and examined PER in response to sucrose and HxA. The synaptobrevin cleavage peptide Tetanus Toxin-Light Chain (TNT) was expressed under the control of Gustatory Receptor or Ionotropic Receptor promoters known to overlap with Gr64f (S1 Table) [19,29,34,35]. Of the 10 GAL4 lines tested, silencing with GAL4 drivers for Gr61a, IR56b, and Gr64f resulted in PER defects to sucrose and HxA. In contrast, silencing Gr64e neurons significantly reduced response to sucrose without affecting response to HxA, and silencing Gr5a, Gr43a, or IR56d neurons significantly reduced PER to HxA without affecting sucrose response.

We chose to further investigate the role of IR56d neurons in FA sensing, since IRs have been found to be involved in detection of non-sugar appetitive tastants, including salt and amino acids [27,36,37]. IR56d neurons have previously been reported to project to an SEZ region that overlaps with sweet-sensing neurons, and a second region that originates in the taste pegs of the proboscis [35]. Outside of sensing carbonation, little is known about ligands that activate the taste pegs or their role in gustation. The sweet-sensing projections of IR56d resembled the region of Gr64f projections that was activated by HxA (Fig 1).

**Fig 2:**
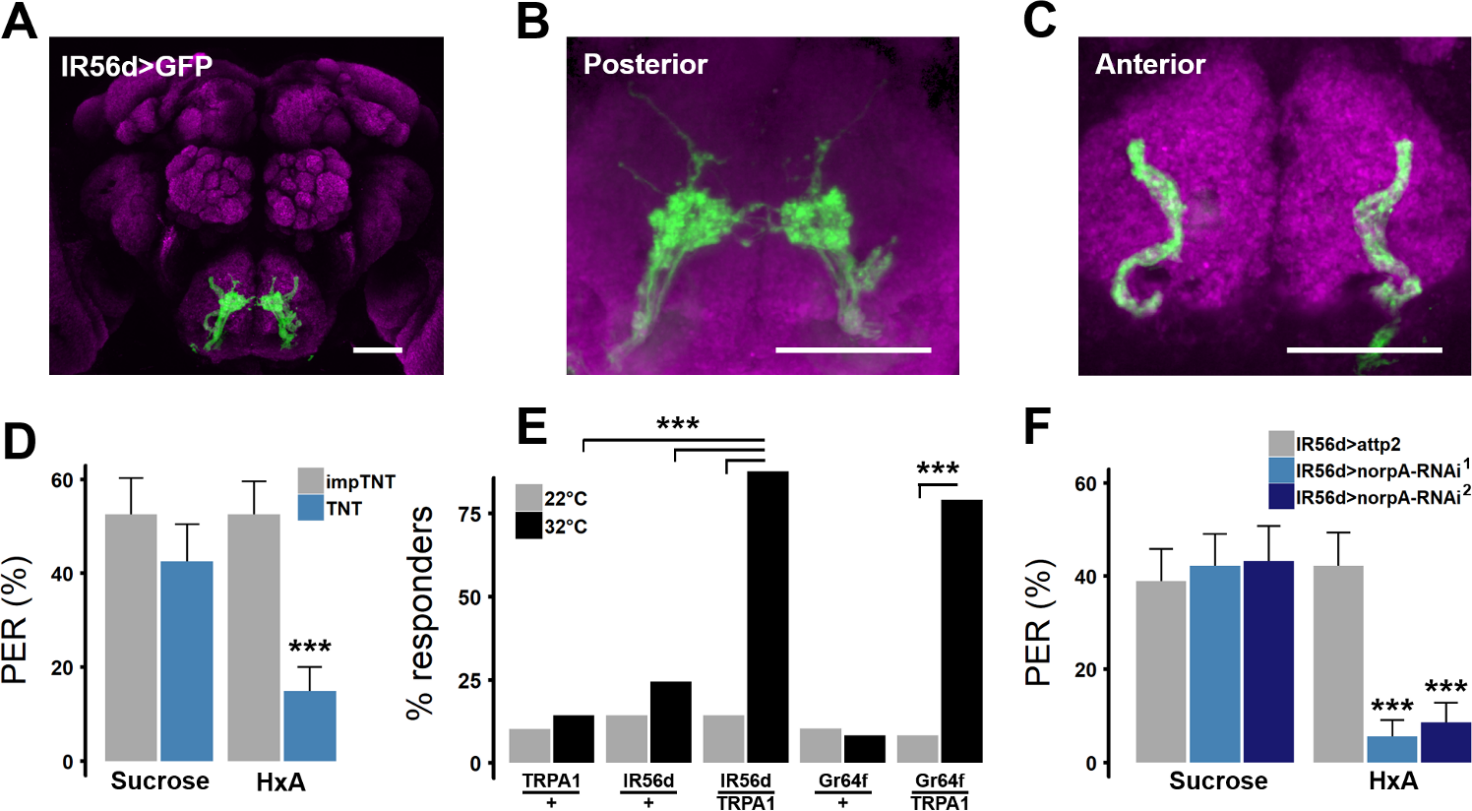
IR56d neurons are necessary and sufficient for PER to FAs. (A) Expression pattern of IR56d neurons visualized with GFP. Distinct regions of projection are seen in (B) posterior and (C) anterior regions of SEZ. Scale bar = 50 μm. (D) Blocking synaptic release by genetic expression of light-chain tetanus toxin (TNT) in IR56d neurons significantly reduces PER to HxA, but not sucrose, compared to control flies expressing an inactive form of tetanus toxin (impTNT). (impTNT n = 26; TNT n = 29). Wilcoxon Rank Sum Test; ***p<0.001. (E) Heat activation of IR56d neurons with TRPA1 induces significant PER compared to either transgene alone, and is comparable to PER induced by activation of Gr64f neurons. (n = 49 for all genotypes). Fisher’s Exact Test with Bonferroni correction for multiple comparisons. (F) Targeted knockdown of *norpA* in IR56d neurons significantly reduces response to HxA, while response to sucrose did not differ from controls flies harboring IR56d-GAL4 alone (N>27 for all genotypes); ***p< 0.001.

Consistent with previous reports, expression of GFP in IR56d neurons (IR56d-GAL4>cd8::GFP) revealed two populations of projections: one innervating the posterior SEZ and previously defined as emanating from labellar bristles, and a second population emanating from the taste pegs innervating the anterior SEZ (Fig 2A–2C) [35]. To determine whether IR56d neurons are required for FA taste we silenced them with TNT and measured PER in response to FA presentation. To control for genetic background and any potential non-specific effects of TNT, we compared PER in flies with silenced IR56d neurons (IR56d-GAL4>UAS-TNT) to flies expressing an inactive variant of TNT (IR56d-GAL4>UAS-impTNT) [34]. Expression of impTNT in IR56d neurons did not affect PER in response to sucrose or HxA, while expression of TNT selectively inhibited HxA response (Fig 2D). Therefore, IR56d neurons are necessary for behavioral responses to HxA, but dispensable for responses to sucrose.

Broad activation of sweet-sensing neurons expressing the trehalose receptor Gr5a induces feeding response in the absence of tastants [16,38,39]. To determine whether activation of IR56d neurons is sufficient to induce PER, we selectively expressed the thermo-sensitive cation channel transient receptor potential A1 (*TRPA1*) in IR56d neurons, or Gr64f neurons as a positive control, and measured heat-induced PER [40,41]. TRPA1 induces neuronal activity at temperatures above 28°C, but has little effect on neuronal activity in flies at 22°C, allowing for thermogenetic modulation of neuronal activity [40,41]. In agreement with previous findings, broad activation of sweet-sensing neurons with Gr64f-GAL4 induced PER (Fig 2E) [26,42,43]. Similarly, PER was significantly greater upon selective activation of IR56d neurons than in control flies harboring UAS-TRPA1 or IR56d-GAL4 transgenes alone (Fig 2E). Therefore, activation of IR56d neurons alone is sufficient to induce PER.

We previously showed that PER response to HxA requires the PLC homolog no receptor potential A (*norpA*) in Gr64f neurons [18]. To determine whether *norpA* is required in IR56d-expressing neurons, we selectively expressed norpA^RNAi^ under control of IR56d-GAL4 and measured PER response to HxA and sucrose. The response to HxA was reduced in experimental flies (IR56d-GAL4>UAS-norpA^RNAi^) using two different norpA^RNAi^ transgenes compared to controls harboring IR56d-GAL4 alone (Fig 2F). In agreement with previous findings examining *norpA* mutants or knock-down of *norpA* in all neurons expressing Gr64f, sucrose sensing was unaffected in IR56d-GAL4>UAS-norpA^RNAi^ flies, indicating that signaling through *norpA* in IR56d neurons is required for response to HxA, but dispensable for sucrose response.

**Fig 3:**
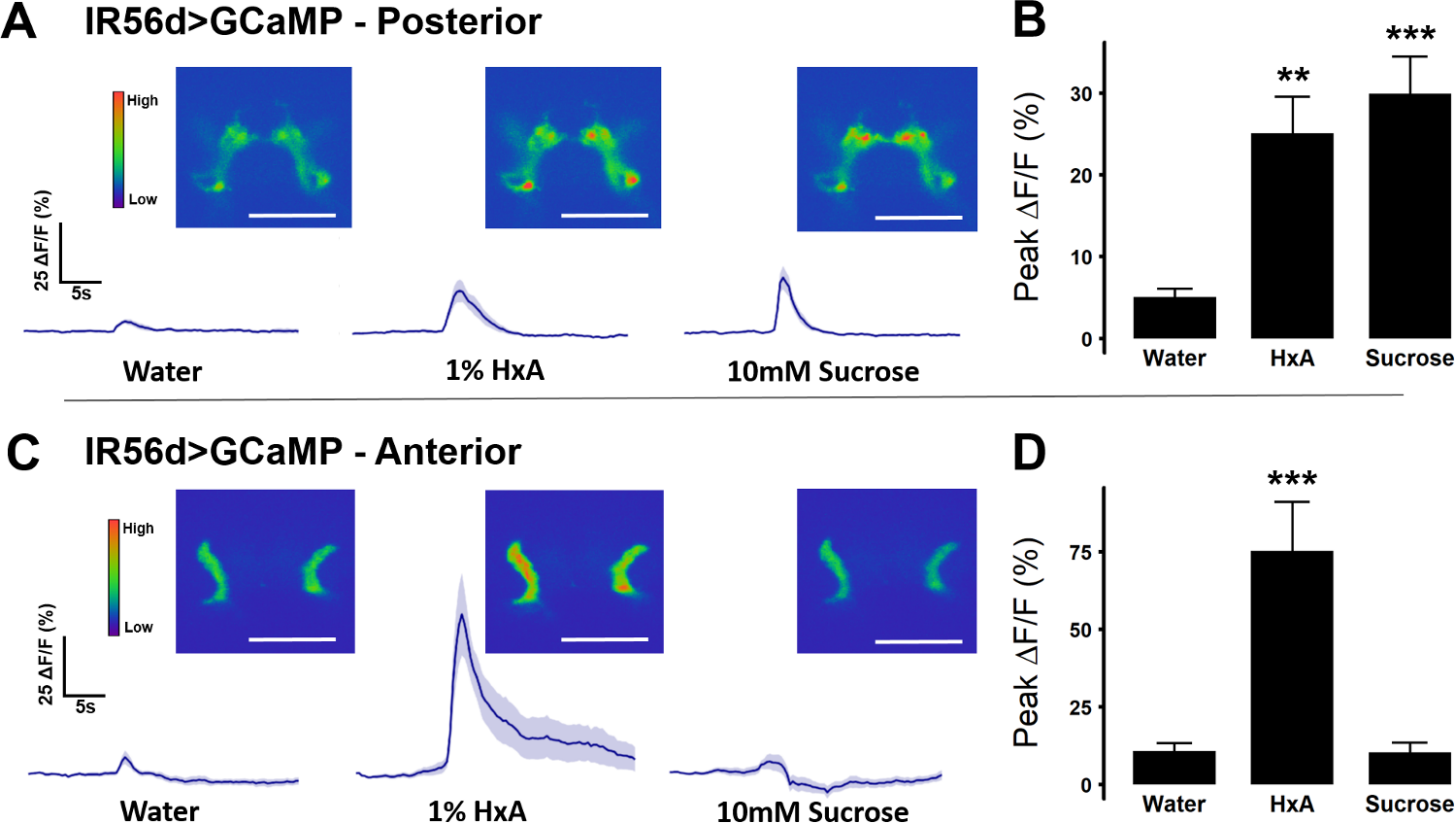
Response to sugar and fatty acid differs in anterior and posterior IR56d projections. (A) Activity traces and representative images of calcium activity in IR56d posterior projections in response to water, 1% HxA, and 10mM sucrose (n = 13 for each tastant). Shaded region of trace indicates +/- SEM (B) Average peak %ΔF/F for data shown in (A). Error bars indicate SEM. One-way ANOVA with Tukey’s HSD; **p< 0.01, ***p< 0.001. (C) Activity traces and representative images of calcium activity in IR56d posterior projections in response to water, 1% HxA, and 10mM sucrose (n = 12 for each tastant). Shaded region of trace indicates +/- SEM (D) Average peak ΔF/F for data shown in (C). Error bars indicate SEM. One-way ANOVA with Tukey’s HSD; ***p< 0.001.

IR56d-expressing neurons project to both taste peg and sweet-sensing regions of the SEZ, and each region can be distinguished anatomically (Fig 2A–2C; [35]). To determine whether sugars and FAs can differentially activate each region, we expressed GCaMP5 in IR56d neurons (IR56d-GAL4>GCaMP5) and measured tastant-evoked activity within anterior and posterior projections. The posterior region, which overlaps with Gr64f neurons, responded to both HxA and sucrose with similar magnitude (Fig 3A and 3B). The anterior projections from the taste pegs, however, responded to HxA, but not sucrose (Fig 3C and 3D). Therefore, the anterior and posterior projecting IR56d neurons are functionally distinguishable by their responsiveness to sucrose.

**Fig 4:**
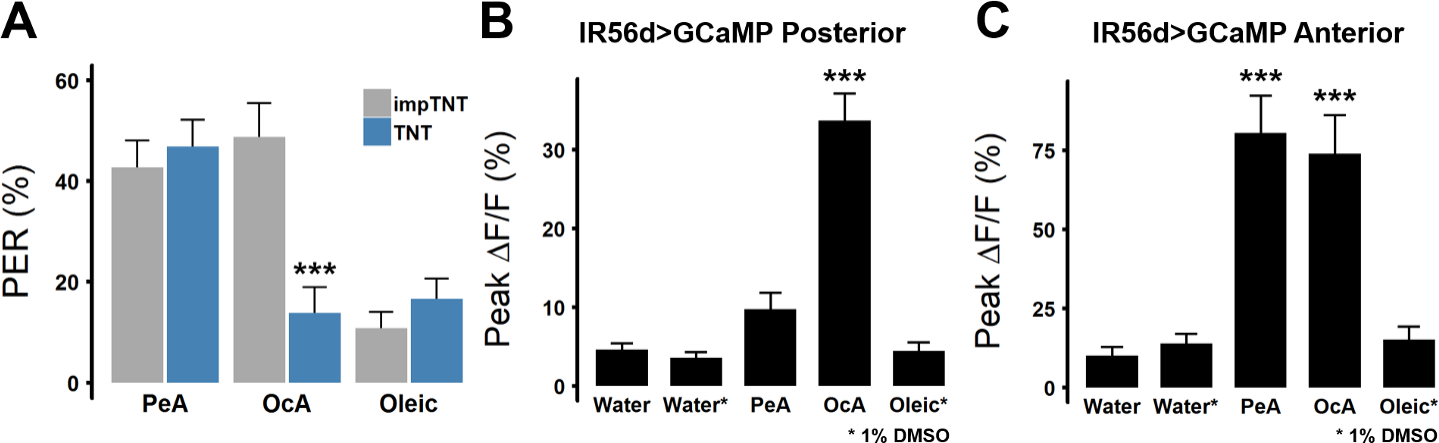
IR56d neurons are selectively responsive to short and medium-chain FAs. (A) PER in response to short-chain pentanoic acid (PeA, n = 46), medium-chain octanoic acid (OcA, n = 26), and long-chain oleic acid (n = 46) in TNT and control impTNT flies. Blocking synaptic release in IR56d neurons with TNT significantly decreases PER to octanoic acid (n = 29), but does not affect PER for pentanoic (n = 42) or oleic acid (n = 42). Wilcoxon Rank-Sum Test; ***p<0.001. (B) Average peak %ΔF/F for the posterior projections of IR56d neurons in response to water, 1% DMSO, pentanoic acid, octanoic acid, and oleic acid (n = 9, 8, 9, 9, 8, respectively), and (C) the anterior projections (n = 9, 7, 9, 9, 7, respectively). Error bars indicate SEM. One-way ANOVA with Tukey’s HSD; ***p<0.001.

Flies exhibit feeding responses to the presentation of several FA classes [18]. It is possible that the IR56d neurons are broadly responsive to different classes of FAs. Alternatively, different classes of FAs may be sensed by independent, or partially overlapping, populations of sensory neurons. To measure the responsiveness of IR56d neurons to different classes of FAs, we first compared behavioral responses of IR56d-silenced flies to short-chain pentanoic acid (5 carbons, 5C), medium-chain octanoic acid (8C), and long-chain oleic acid (18C). As compared to control IR56d>impTNT flies, IR56d>TNT flies exhibited reduced PER to octanoic acid, but retained PER to pentanoic acid (Fig 4A), suggesting that PER to short-chain FAs is not dependent on IR56d neurons. Oleic acid did not elicit strong PER in control flies, suggesting flies do not respond to at least some long-chain FAs. We directly examined IR56d neuron responsiveness to different FAs with *in vivo* Ca^2+^ imaging in IR56d-GAL4>GCaMP5 flies. Octanoic acid activated both the posterior and anterior IR56d projections, while pentanoic acid selectively activated anterior IR56d projections (Fig 4B and 4C). Oleic acid, which did not induce PER, did not activate IR56d projections in either regions (Fig 4B and 4C). Together, these findings reveal that IR56d neurons respond to short and medium-chain FAs, and further, that sub-populations localized by SEZ projections have distinct FA response profiles.

**Fig 5:**
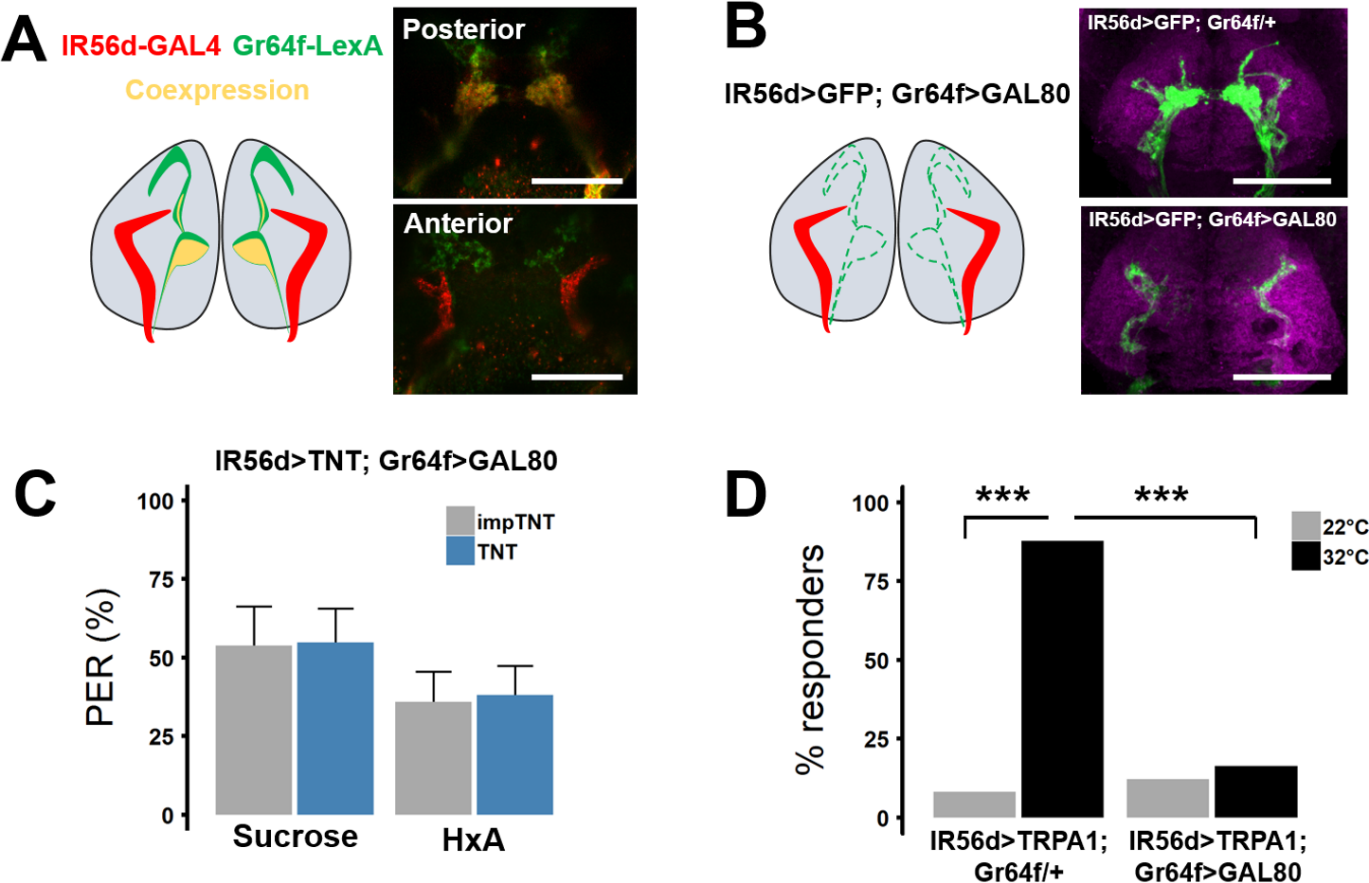
IR56d anterior projections are dispensable for PER to FAs. (A) IR56d and Gr64f neurons visualized with IR56d-GAL4 driving RFP and Gr64f-LexA driving GFP. Co-localization is detected in the posterior projections, but is minimal in anterior projections. (B) Driving the GAL4 repressor Gal80 with Gr64f-LexA limits GFP expression to the non-overlapping IR56d anterior projections. (C) Restricting TNT expression to the non-overlapping anterior projection neurons does not significantly impact PER to sugar or HxA (impTNT n = 23; TNT n = 22). Error bars indicate SEM. Wilcoxon Rank Sum Test Sucrose: *p*>0.98; HxA: *p*>0.96. (D) Restricting TRPA1 expression to the non-overlapping neurons abolishes PER compared to control in which TRPA1 is driven in all IR56d neurons. Fisher’s Exact Test with Bonferroni correction for multiple comparisons; ***p<0.001.

The findings that silencing of IR56d or Gr64f neurons abolishes PER to hexanoic and octanoic acids raises the possibility that neurons co-expressing both receptors are required for FA response (Fig 5A). To validate co-expression of IR56d and Gr64f, we used the LexA system to label Gr64f neurons (Gr64f-LexA>LexAOp-CD8:GFP) and the GAL4 system to label IR56d neurons (IR56d-GAL4>UAS-RFP) (Fig 5A). Examining SEZ projections revealed co-localization within the posterior SEZ, with no co-localization detected in the anterior SEZ, suggesting the posterior IR56d neurons co-express Gr64f and IR56d. To determine whether the IR56d/Gr64f co-expressing neurons are required for FA taste, we used intersectional strategies to selectively silence anterior IR56d neurons of the taste pegs. Specifically, we repressed TNT expression in IR56d/Gr64f co-expressing neurons using Gr64f-LexA to drive expression of the GAL80 repressor (IR56d-GAL4>UAS-TNT; Gr64f-LexA>LexAop-GAL80) (Fig 5B). Selectively silencing IR56d neurons that do not overlap with Gr64f did not affect PER to HxA or sucrose compared to impTNT controls, suggesting the taste peg neurons are dispensable for the reflexive feeding response to FAs (Fig 5C). Flies lacking Gr64f-LexA, but still harboring a copy of LexAop-GAL80 (IR56D-GAL4>UAS-TNT; LexAop-GAL80/+), showed reduced PER as expected (S1 Fig).

To test if the IR56d taste peg neurons are sufficient to induce PER, we measured heat-induced PER in flies containing TRPA1 in the restricted expression pattern (IR56d-GAL4>UAS-TRPA1; Gr64f-LexA>LexAop-GAL80). Heat-induced PER was significantly reduced in flies expressing TRPA1 in only the IR56d taste peg neurons compared to control flies that lacked GAL80, and thus expressed TRPA1 in all IR56d neurons (Fig 5D). Together these results suggest IR56d neurons that do not overlap with Gr64f neurons are dispensable for PER in response to FAs.

**Fig 6:**
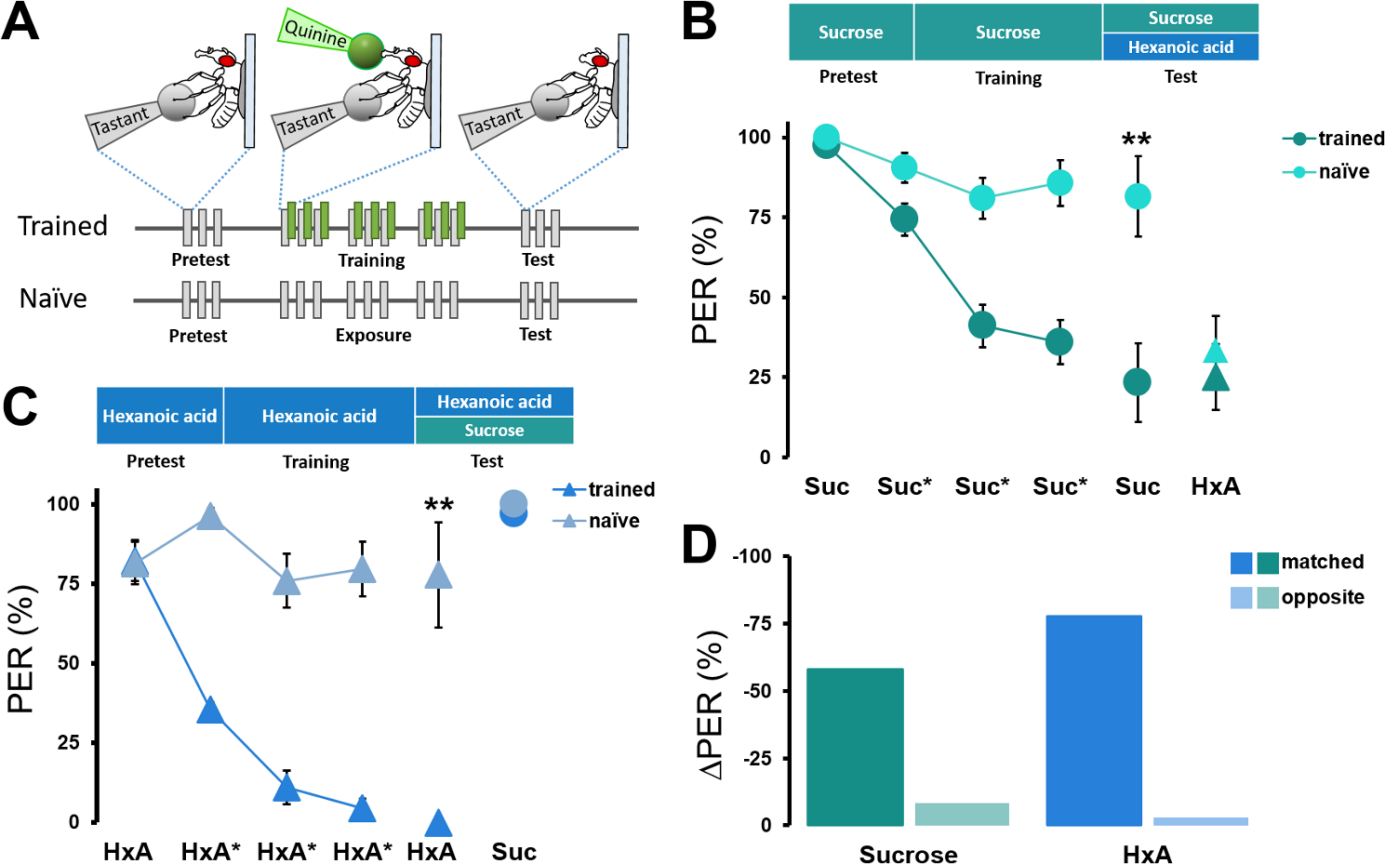
*Drosophila* discriminates between sucrose and HxA. (A) Taste memory protocol to determine sucrose and FA discrimination. Flies are trained by pairing HxA or sucrose on tarsi with quinine on proboscis. PER in response to HxA and sucrose is then tested following training to sucrose or HxA in the absence of quinine. In control experiments (naïve), the same procedure is followed, but quinine is not applied to the proboscis. (B) The pairing of sucrose and quinine (dark green circles) results in a significant reduction in PER across all three training trials compared to unpaired naïve flies (light green circles). PER response to sucrose in the test is significantly lower in trained flies compared to naïve flies (n = 7, 11), but no differences in response to HxA (triangles) is detected between the experimental and naïve groups (n = 11, 12). Kruskal-Wallis Test followed by Dunn’s Test (control: w^1118^); **p<0.01. (C) The pairing of HxA and quinine (dark blue triangles) results in a significant reduction in PER across all three training trials compared to unpaired naïve flies (light green triangles). The test PER response to HxA alone is significantly lower in trained flies compared to naïve flies (n = 13, 16), but no differences in response to sucrose (circles) is detected between the groups (n = 12, 12). Kruskal-Wallis Test followed by Dunn’s Test (control: w^1118^); **p<0.01. (D) Percent suppression of PER reveals that flies trained and tested to the same tastant (either sucrose or HxA) show significantly reduced PER compared to flies trained and tested with different tastants.

Although both sugars and FAs induce feeding behavior, it is unclear whether flies can qualitatively differentiate between these two classes of appetitive tastants. We have developed an assay in which an appetitive tastant is applied to the tarsi, paired with a bitter quinine application to the proboscis, and the suppression of PER in subsequent responses to the appetitive tastant is then measured [39,44]. To determine whether flies can differentiate between sugars and FAs, we applied sucrose (conditioned stimulus) to the tarsi followed immediately by quinine application (unconditioned stimulus) to the proboscis. Following three training trials, memory was tested by application of either sucrose or HxA to the tarsi, in the absence of quinine, and measuring PER (Fig 6A). We then performed reciprocal experiments in which flies were trained with HxA and tested for PER to HxA or sucrose.

As previously reported, pairing sugar with quinine significantly reduced PER over the course of three training trials compared to flies offered only sugar without quinine (Fig 6B) [45,46]. This suppression of PER persisted in the testing phase where quinine is not presented (Fig 6B). On the contrary, there were no differences in PER to HxA between flies repeatedly provided sucrose in the absence of quinine and flies trained with sucrose-quinine pairing, indicating that the aversive taste memory formed to sucrose is not generalized to HxA. Conversely, the pairing of HxA and quinine resulted in PER suppression to HxA that was not generalized to sucrose (Fig 6C). We did observe a reduction in total PER response to HxA when flies had previously received sucrose tastant (Fig 6B and 6C). This occurred when prior stimulation was paired with quinine or unpaired, suggesting it is independent of memory formation and likely due to the comparative difference in salience between the two tastants. Quantification of the percentage reduction in PER revealed that the ‘matched’ groups, where quinine is paired with the tastant that is later tested, suppressed PER by 79–94%, while there was no significant PER suppression in the ‘opposite’ group, where the quinine-paired tastant and the tested tastant were different (Fig 6D). This reciprocal discrimination between sucrose and HxA is different from the unilateral discrimination between two sugars reported previously [44] and indicates that flies can discriminate between sucrose and HxA based on their identity. Thus, sugars and FAs act as independent taste modalities in flies.

## Discussion

### Localization of fatty acid-sensitive neurons

Sweet-sensing neurons in *Drosophila* have been broadly classified as those responding to sugars and other attractive tastants such as glycerol and amino acids [16,27,47]. The findings presented here further localize the reflexive feeding response to hexanoic and octanoic acids, both medium-chain FAs, to a small population of FA-responsive taste neurons that partially overlap with sweet-sensing neurons. We have previously shown that genetic silencing of most sweet-sensing neurons using Gr64f-GAL4 abolished FA response, suggesting that these neurons detect sugars and FAs [18]. In flies, some subpopulations of Gr64f neurons are selectively sensitive to certain tastants including a Gr64e population that is sensitive to glycerol [47] and a Gr5a subset that is sensitive to trehalose [16]. To localize the Gr64f neurons responsible for FA taste, we conducted a targeted screen and silenced neurons that are believed to overlap with Gr64f neurons, which led us to study the IR56d population of neurons. Silencing IR56d neurons appears to selectively disrupt HxA response without affecting response to sucrose, supporting the notion that independent mechanisms within the Gr64f population mediate responses to sugars and FAs.

It is possible that FA-sensitive neurons are broadly tuned to FAs or selectively respond to distinct classes of FAs. Our Ca^2+^ imaging experiments indicate that IR56d neurons are responsive to medium-chain HxA (C6, saturated) and octanoic acid (C8, saturated) in both anterior and posterior regions if the SEZ, and to short-chain pentanoic acid (C5, saturated), but only in the anterior projections. We do not find IR56d neurons responsive to long-chain oleic acid (C18, mono-unsaturated). These findings are supported by behavioral data revealing that flies exhibit PER in response to pentanoic acid, HxA, and octanoic acid, but not oleic acid. Therefore, it seems likely that flies are strongly responsive to short/medium-chain FAs, but are less responsive to long-chain and/or unsaturated FAs. The finding that PER elicited by pentanoic acid occurs even when Ir56d neurons are genetically silenced suggests independent populations of taste neurons drive PER in response to short-chain and medium-chain FAs. Further, IR56d neurons may be activated by long-chain FAs that were not tested, and these could modulate feeding response and induce PER. Nevertheless, the findings presented here reveal specificity for medium-chain FAs within a defined population of taste neurons.

Many of the neurons identified by IR56d expression express multiple taste receptors including IR56d, Gr64f and Gr5a. These neurons likely express many additional candidate taste receptors, and future studies are needed to identify the receptor(s) that are responsive to FAs. IRs are related to ionotropic Glutamate receptors and respond to diverse tastants and odorants, making them excellent candidates for detecting FAs. [48,49]. While IR56d remains an excellent candidate, it will be necessary to examine potential IR co-receptors that may be critical for IR-dependent sensation. For example, IR25a is relatively broadly expressed and likely functions as a co-receptor for numerous IR-dependent sensory processes including temperature sensing and hygrosensation [37,50–52]. It is possible that multiple IRs are required for FA taste, with some acting as co-receptors and others detecting specific FAs. While future work is required to identify the molecular bases for FA taste, the identification of FA sensitivity in IR56d neurons provides a system to interrogate the cellular mechanisms of FA taste.

### Discrimination between attractive substances

The PER response induced by two different medium-chain FAs, hexanoic and octanoic acids, suggests they may be part of *Drosophila melanogaster* diet. Typical dietary fats, including many plant based oils, such as coconut oil, are rich in longer-chain FAs including palmitic acid, oleic acid and linoleic acid [53]. However, medium-chain FAs are present in fermenting fruits such as guava and also in pollen [54,55]. Moreover, the medium-chain FAs (mostly C6-C10) are excreted by yeast during fermentation, possibly helping with finding yeast-rich feeding substrates, raising the possibility that flies have developed a response to FAs in order to locate suitable fermented food sources [56]. Further, we have previously shown that a diet of HxA alone is sufficient for survival [18]. Therefore, it is possible that FA attraction evolved to promote consumption of calorically rich fermenting fruits consumed by *Drosophila*.

The use of sucrose and HxA in an aversive taste memory paradigm reveals flies can discriminate between these attractive tastants. Sugars induce broad activation of Gr64f neurons, while the activation induced by HxA appears more restricted, and therefore it is possible that differences in activation allow for differentiation [57]. Alternatively, we find that HxA also activates anterior-projecting IR56d neurons that emanate from the taste pegs and do not co-express Gr64f, raising the possibility that differential response of these neurons to sucrose and FAs allows discrimination. Considering the different biochemical pathways involved in sugar and FA sensing [18], their identity may also be coded by unique temporal and spatial dynamics of sensory neuron activation [15,58,59]. Differences in activation are suggested to provide a mechanism for olfactory discrimination within defined neural populations, and it is possible that similar mechanisms are utilized for attractive tastants [60]. In *Drosophila*, attractive tastants have been found to induce a wide range of excitatory responses ranging from acute to prolonged firing [28,61], providing a potential mechanism for discrimination. While the sensillar response to FAs has not been reported, the differences in Ca^2+^ response to sugar or HxA presentation within the SEZ suggest differences in temporal activation.

Our findings reveal the population of IR56d neurons that innervate the anterior SEZ, which emanate from the taste pegs, are dispensable for PER in response to FAs. However, it is possible these neurons are still involved in discrimination between FAs and sugars. These neurons are not responsive to sucrose, therefore distinct anatomical activation may account for the gustatory discrimination between attractive substances. The taste pegs have previously been implicated in sensing non-sugar attractive tastants including polyamines and carbonation, raising the possibility that these neurons are responsive to multiple taste modalities [25,26]. Selectively silencing the IR56d taste peg neurons and measuring discrimination between FAs and sugars may determine whether distinct classes of IR56d neurons mediate taste feeding response and taste discrimination.

We find that flies can discriminate between sugars and FAs, but it is not known whether they can discriminate qualitatively between different classes of FAs. A previous study examining discrimination between different sugars found that flies are unable to discriminate based on quality, but could discriminate based on perceived palatability [44]. Here, we find that pentanoic acid elicits a PER response that is independent of IR56d neurons. The findings, coupled with evidence that distinct populations of neurons respond to FAs from different classes, raise the possibility that flies may discriminate between FAs based on the identity of neurons activated by each FA, or classes of FAs.

### Role of Phospholipase C in FA taste

We previously reported that PLC signaling in sweet-sensing Gr64f neurons is required for FA taste [18]. Flies with mutation or knockdown of the PLC-ß ortholog *norpA* do not respond to HxA or octanoic acid but respond normally to sugars, revealing independent intercellular signaling mechanisms likely underlie response to FAs and sugars [18,62]. We find that knockdown of *norpA* in IR56d neurons abolishes FA taste without disrupting the taste of sucrose. These findings phenocopy *norpA* mutants and broad knockdown of *norpA* in Gr64f neurons, fortifying the notion that PLC signaling is selectively required for FA taste [18]. Testing the response of *norpA* deficient flies to FAs that are sensed independently of IR56d will inform whether PLC is more generally required for FA taste, or is only specific to medium-chain FAs detected by IR56d neurons.

### Higher order neurons involved in sensory discrimination

While taste coding within the SEZ has been extensively investigated, less is known about the higher order circuits governing taste. Sweet-sensing neurons connect to the antennal mechanosensory and motor center (AMMC) and downstream PAM dopamine neurons that are activated by sugar [38,63]. In addition, a separate population of dopamine neurons, the PPL1 cluster, is required for olfactory appetitive memory and taste aversive conditioning [64–66]. To date, higher order neurons responsive to FA taste have not been identified. It is possible that sugar and FA taste signal through shared higher order dopamine neurons or, alternatively, each taste modality may activate distinct populations of higher order neurons that convey valence to the mushroom bodies, the memory and sensory integration center in insects [67–69].

While both sugars and FAs activate shared neurons, the ability to discriminate between these tastants provides a model for investigating sensory discrimination. There is growing evidence of multimodal coding within *Drosophila* sensory neurons, and in downstream targets. Flies harboring only a single functional type of olfactory receptor neurons are able to discriminate between odorants, presumably due to differences in temporal activation between the odorants [70]. Further, in the larval taste system, a single gustatory receptor neuron is responsive to both attractive and aversive compounds, and mediates the integration of these competing stimuli [71]. In addition to integration of distinct cues by the sensory system, the *Drosophila* mushroom bodies, and courtship circuitry integrate complex sensory cues within the brain [72,73]. Future studies on how the central brain processes sugar and FA taste will help elucidate mechanisms of discrimination between sugars and FAs.

### *Drosophila* as a model for investigating FA taste in mammals

Despite the role of FAs in promoting feeding, surprisingly little is known about how FAs promote taste in any model system. Fats contain many sensory cues and separating the taste of fat per se, from other cues such as texture, viscosity and smell is a particular challenge in mammals [74]. A number of studies have implicated the lipid binding protein CD36 as contributing to FA taste. CD36 is expressed in gustatory oral tissue, and appears to be selectively involved in FA taste [75]. CD36 knockout animals show no preference for FAs but retain preference for sugars [76]. The *Drosophila* homolog of CD36, Sensory neuron membrane protein 1, is expressed in the olfactory system and required for sensation of the pheromone cis-vaccenyl acetate [77], and therefore is unlikely to mediate FA taste. Additionally, a number of FA-binding GPCRs are expressed in taste cells, but their role in FA taste has not been identified. The ability to selectively manipulate and ablate defined classes of sensory neurons in *Drosophila* allows for the disambiguation of taste from other sensory processes [78]. Identifying FA receptors and neural circuitry mediating FA taste and discrimination will provide a framework for investigating similar processes in mammalian systems.

### Conclusions

Taken together, this study provides insight into the coding of FAs within the fly gustatory system. Our results reveal a population of sweet-sensing neurons that are tuned for medium-chain FAs, but not short- or long-chain FAs. Further, the finding that flies are capable of discriminating between FAs and sugars suggests coding differences, either spatial or temporal neuronal activation, and provides a mechanism to distinguish between tastants of the same valence. Understanding how FAs are coded within the fly brain provides a model for understanding taste in more complex systems and will offer insight into generalizable mechanisms for taste discrimination.

## Materials and methods

### *Drosophila* maintenance and fly stocks

Flies were grown and maintained on standard food (New Horizon Jazz Mix, Fisher Scientific). Flies were maintained in incubators (Powers Scientific; Dros52) at 25°C on a 12:12 LD cycle, with humidity set at 55–65%. The background control line used in this study is the *w*^1118^ fly strain unless otherwise noted. The following fly strains were ordered from The Bloomington Stock Center, UAS-impTNT (28840), UAS-TNT (28838), UAS-TRPA1 (26263); UAS-GFP (32186); UAS-GCaMP5 (42037); UAS-RFP/LexAop-GFP (32229); Gr43a-GAL4 (57637), Gr5a-GAL4 (57591), Gr61a-GAL4 (57658), Gr64a-GAL4 (57662), Gr64c-GAL4 (57663), Gr64d-GAL4 (57665), Gr64e-GAL4 (57666), Gr64f-GAL4 (57668), IR56b-GAL4 (60706), IR56d-GAL4 (60708), LexAop-Gal80 (32213), norpA-RNAi^1^ (31113), and norpA-RNAi^2^ (31197). Gr64f-LexA was a kind gift from H. Tanimoto and previously described in [19]. Seven to nine day old mated female flies were used for all experiments in this study, except when noted.

### Proboscis extension reflex

For all experiments, one-week-old flies were fasted for 48 hours prior to testing. For the initial screen using TNT, and specific testing of tarsal response, PER was measured by applying tastant to the tarsi, as previously described [18]. For all other PER experiments, including validation of IR56d phenotypes, tastant was applied to the proboscis to match behavioral results with functional imaging. Flies were anesthetized on CO_2_, mounted in a pipette tip so that their head and proboscis, but not tarsi, were exposed, and allowed to acclimate for a minimum of 30 minutes prior to testing. Flies that did not stop responding to water within 5 minutes were discarded. A small KimWipe (Kimberley Clark) wick saturated with tastant was manually applied to the tip of the proboscis for 1–2 seconds and proboscis extension reflex was monitored. Only full extensions were counted as a positive response. Each tastant was presented three times, with 10 seconds between each trial. Between tastant trials, the proboscis was washed with water and flies were allowed to drink. PER response was calculated as a percentage of proboscis extensions to total number of tastant stimulations. For experiments examining the effects of TRPA1 activation on PER, flies were mounted on a microscope slide using nail polish as described previously [18]. Flies were then placed on a heat plate heated to 34°C and video of activity was recorded for 1 minute. The number of flies for each genotype showing PER within the trial period was counted and the percentage of flies showing PER calculated.

### *In vivo* functional imaging

Flies were anaesthetized on ice and placed in a small chamber with the head and proboscis accessible. A small hole was cut in tin foil and fixed to the stage leaving a window of cuticle exposed, then sealed using dental glue (Tetric EvoFlow–Ivoclar Vivadent). The proboscis was extended and a small amount of dental glue was used to secure it in place, ensuring the same position throughout the experiment.

The cuticle and connective tissue was dissected to expose the SEZ, which was bathed in artificial hemolymph (140mM NaCl, 2mM KCl, 4.5mM MgCl2, 1.5mM CaCl2, and 5mM HEPES-NaOH with pH = 7.0). Mounted flies were placed under a confocal microscope (Nikon A1) and imaged using a 25x water-dipping objective lens. The pinhole was opened to allow a thicker optical section to be monitored. Recordings were taken at 4Hz with 256 resolution. Tastants were delivered to the proboscis for 1–2 seconds with a KimWipe wick operated by micromanipulator (Narishige, GJ-1). For analysis, regions of interest were drawn manually, taking care to capture the same area between control and experimental. Baseline fluorescence was recorded over 5 frames, 10 seconds prior to tastant application. %ΔF/F was calculated for each frame as (fluorescence—baseline fluorescence)/baseline fluorescence * 100. Average fluorescence traces were created by taking the average and standard error of %ΔF/F for all samples per frame.

### Immunohistochemistry

Fly brains were dissected in ice-cold PBS and fixed in 4% formaldehyde, PBS, 0.5% Triton-X 100 for 30 minutes. Brains were rinsed 3X with PBS, Triton-X for 10 min and incubated overnight at 4°C in NC82 (Iowa Hybridoma Bank [79]). The brains were rinsed again in PBS-TritonX, 3X for 10 minutes and placed in secondary antibodies (Donkey anti-Mouse 555; Life Technologies) for 90 minutes at room temperature. The brains were mounted in Vectashield (VectorLabs) and imaged on confocal microscope. Brains were imaged in 2um sections and are presented as the Z-stack projection through either the entire brain, or anterior and posterior regions of IR56d projections in the SEZ.

### Aversive taste memory

PER induction was performed in one week old mated females as described previously [5, 16]. Flies were collected and placed on fresh food for 24 hours and then fasted for 48 hours in vials containing wet KimWipe paper. Flies were later anaesthetized on CO_2_ pad and glued using nail polish (Cat#72180, Electron Microscopy Science) by their thorax and wing base on a microscopy slide and left to recover in a humidified box for 3-6h prior to experiments. For experiments, the slide was mounted vertically under a dissecting microscope (Olympus SZX12) during which PER was observed. Flies were satiated with water before and during the experiment. Flies that did not initially satiate within 5 minutes were excluded from conditioning. A 1ml syringe (Tuberculine, Becton Dickinson & Comp) with 200uL pipette tip attached was used for tastant presentation. We used purified water, 10mM and 1000mM sucrose, 0.4% hexanoic acid and 10mM quinine hydrochloride solutions. The protocol was adapted from [39]. Briefly, for pretest, each fly was given 10mM sucrose or 0.4% HxA on their tarsi three times with 20 second inter-trial interval and the number of full proboscis extensions was recorded. During training, the same stimulation as before was followed by placing a droplet of 10mM quinine on the extended proboscis, during which flies were allowed to drink for up to 2 seconds or until they retracted their proboscis. After each session, the tarsi and proboscis were washed with water and flies were allowed to drink to satiation. After training, flies were tested with either that same substance without quinine or with the untrained substance (matched or opposite trained groups). An independent group of flies were measured as described above but quinine was never presented (naïve groups). At the end of each experiment, flies were given 1000mM sucrose to check for retained ability of PER and non-responders were excluded [11].

### Reagents

Sucrose was purchased from Fisher Scientific (FS S5-500). All other tastants were purchased from Sigma Aldrich. Sucrose, hexanoic acid (SA 153745), octanoic acid (SA C2875), pentanoic acid (SA 240370) and quinine hydrochloride (SA 145904) were diluted in H_2_0. Oleic Acid (SA O1008) was diluted in 1% DMSO (Sigma).

### Statistical analysis

All statistical tests were performed in R. For normally distributed data, Welch’s t-test or ANOVA with Tukey’s post-hoc comparison were performed. For data that did not fit a normal distribution, Wilcoxon Rank-Sum or Kruskal-Wallis with Dunn’s post-hoc tests were used. Fisher’s Exact Test was used for binary categorical data. For all tests with multiple comparisons, a Bonferroni *p*-value adjustment was performed.

## Supporting information

S1 Table A neuronal silencing screen for taste neurons sensitive to HxA. (A) Data for PER in response to 100mM sucrose in flies with silenced populations of gustatory neurons by driving expression of TNT with the indicated. The ‘normalized’ column represents PER of the experimental divided by the PER of the control line, w^1118^. (B) Data for PER in response to 1% HxA. Kruskal-Wallis Test followed by Dunn’s Test (control: w^1118^) with Bonferroni correction for multiple comparisons; *p<0.05, **p<0.01, ***p<0.001.
(PDF)

S1 Fig Flies continue to suppress PER in response to FAs in absence of Gr64f to drive LexAop-Gal80. PER of flies expressing either impTNT or TNT in IR56d-expressing neurons (n = 31 for both groups). Without Gr64f-LexA to drive the LexAop-GAL80, as in Fig 5C, TNT is expressed in all IR56d-expressing neurons and PER to HxA is suppressed. Wilcoxon Rank Sum Test; ***p<0.001.
(PDF)
