# Supplementary material for: A subset of sweet-sensing neurons identified by IR56d are necessary and sufficient for fatty acid taste
Source: PLoS Genet. 2017 Nov 9;13(11):e1007059. doi: 10.1371/journal.pgen.1007059 (PMC5697886; doi:10.1371/journal.pgen.1007059)
Supplement: S1 Table — (A) Data for PER in response to 100mM sucrose in flies with silenced populations of gustatory neurons by driving expression of TNT with the indicated. The ‘normalized’ column represents PER of the experimental divided by the PER of the control line, w1118. (B) Data for PER in response to 1% HxA. Kruskal-Wallis Test followed by Dunn’s Test (control: w1118) with Bonferroni correction for multiple comparisons; *p<0.05, **p<0.01, ***p<0.001. (PDF) [file pgen.1007059.s001.pdf]

A **100mM Sucrose**

GAL4	PER (%)	SE	n	normalized	p
w1118 control	100.00	0.00	35	1.00	-
Gr64a	93.33	6.67	10	0.93	-
Gr64c	100.00	0.00	15	1.00	-
Gr64d	97.22	2.78	12	0.97	-
Gr64e	20.00	9.09	15	0.20	***
Gr64f	5.69	1.98	41	0.06	***
Gr5a	79.05	5.65	35	0.79	-
Gr61a	26.19	10.59	14	0.26	***
Gr43a	95.37	3.01	36	0.95	-
Ir56b	41.90	6.16	35	0.42	***
Ir56d	92.59	4.48	27	0.93	-

B **1% HxA**

GAL4	PER (%)	SE	n	normalized	p
w1118 control	47.83	8.85	35	1.00	-
Gr64a	50.00	8.42	22	1.05	-
Gr64c	66.67	7.97	15	1.39	-
Gr64d	62.12	8.29	22	1.30	-
Gr64e	24.24	6.53	33	0.51	-
Gr64f	1.63	1.63	41	0.03	***
Gr5a	1.80	1.26	37	0.04	***
Gr61a	0.00	0.00	14	0.00	***
Gr43a	14.81	5.55	36	0.31	**
Ir56b	3.81	1.82	35	0.08	***
Ir56d	11.11	4.71	27	0.23	**
