# Supplementary figures and images for: A subset of sweet-sensing neurons identified by IR56d are necessary and sufficient for fatty acid taste
Source: PLoS Genet. 2017 Nov 9;13(11):e1007059. doi: 10.1371/journal.pgen.1007059 (PMC5697886; doi:10.1371/journal.pgen.1007059)

Ir56d>TNT; LexAop-Gal80/+

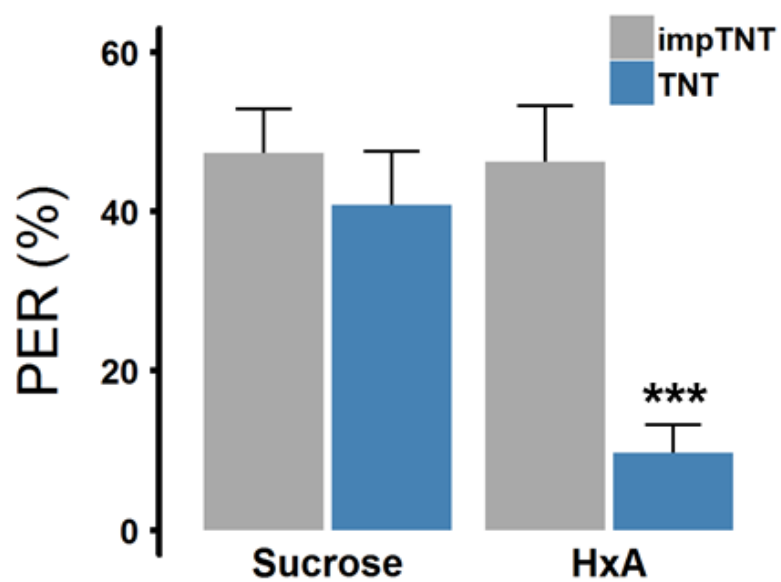

Supplement: S1 Fig — PER of flies expressing either impTNT or TNT in IR56d-expressing neurons (n = 31 for both groups). Without Gr64f-LexA to drive the LexAop-GAL80, as in Fig 5C, TNT is expressed in all IR56d-expressing neurons and PER to HxA is suppressed. Wilcoxon Rank Sum Test; ***p<0.001. (PDF) [file pgen.1007059.s002.pdf]
